# External Validation of the American Heart Association PREVENT Cardiovascular Disease Risk Equations

**DOI:** 10.1001/jamanetworkopen.2024.38311

**Published:** 2024-10-11

**Authors:** Britton Scheuermann, Alexandra Brown, Trenton Colburn, Hisham Hakeem, Chen Hoe Chow, Carl Ade

**Affiliations:** 1 College of Health and Human Sciences, Kansas State University, Manhattan; 2 Department of Biostatistics and Data Science, University of Kansas Medical Center, Kansas City; 3 Department of Physician Assistant Studies, Kansas State University, Manhattan; 4 Cotton O’Neil Heart Center, Stormont Vail Health, Topeka, Kansas; 5 Johnson Cancer Research Center, Kansas State University, Manhattan

## Abstract

**Importance:**

The American Heart Association’s Predicting Risk of Cardiovascular Disease Events (PREVENT) equations were developed to extend and improve on previous cardiovascular disease (CVD) risk assessments for the purpose of treatment initiation and patient-clinician communication.

**Objective:**

To assess prognostic capabilities, calibration, and discrimination of the PREVENT equations in a study sample representative of the noninstitutionalized, US general population.

**Design, Setting, and Participants:**

This prognostic study used data from the National Health and Nutrition Examination Survey (NHANES) 1999 to 2010 data cycles. Participants included adults for whom 10-year follow-up data were available. Data curation and analyses took place from December 2023 through May 2024.

**Main Outcomes and Measures:**

Primary measures were risk estimated by the PREVENT equations, as well as risk estimates from the previous Pooled Cohort Equations (PCEs). The primary outcome was composite CVD-related mortality at 10 years of follow-up. Additional analyses compared the PREVENT equations against the PCEs. Model discrimination was assessed with receiver-operator characteristic curves and Harrell C statistic from proportional hazard regression; model calibration was determined as the slope of predicted versus observed risk.

**Results:**

The study cohort, accounting for NHANES complex survey design, consisted of 172.9 million participants (mean age, 45.0 years [95% CI, 44.6-45.4 years]; 52.1% women [95% CI, 51.5%-52.6%]). In analyses adjusted for the NHANES survey design, a 1% increase in PREVENT risk estimates was statistically significantly associated with increased CVD mortality risk (hazard ratio, 1.090; 95% CI, 1.087-1.094). PREVENT risk scores demonstrated excellent discrimination (C statistic, 0.890; 95% CI, 0.881-0.898) but moderate underfitting of the model (calibration slope, 1.13; 95% CI, 1.06-1.21). PREVENT risk models performed statistically significantly better than the PCEs, as assessed by the net reclassification index (0.093; 95% CI, 0.073-0.115).

**Conclusions and Relevance:**

In this prognostic study of the PREVENT equations, PREVENT risk estimates demonstrated excellent discrimination and only modest discrepancies in calibration. These findings provided evidence supporting utilization of the PREVENT equations for application in the intended population as suggested by the American Heart Association.

## Key Points

**Question:**

What are the prognostic capabilities, calibration, and discrimination of the American Heart Association’s Predicting Risk of Cardiovascular Disease Events (PREVENT) equations in a cohort representative of the US population?

**Findings:**

In this prognostic validation study with data representing 172.9 million participants, the PREVENT equations demonstrated excellent discrimination for predicting fatal events over a 10-year follow-up. Furthermore, discrimination and calibration of the PREVENT equations were significantly improved over the previous criterion-standard approach, the Pooled Cohort Equations.

**Meaning:**

The American Heart Association PREVENT equations demonstrated moderate-to-strong discrimination and calibration for predicting cardiovascular disease risk in the present cohort, providing evidence supporting the validity of using these equations in clinical settings for the intended population.

## Teaser

This prognostic study assesses the prognostic capabilities, calibration, and discrimination of the American Heart Association’s Predicting Risk of Cardiovascular Disease Events (PREVENT) equations using data from the National Health and Nutrition Examination Survey 1999 to 2010 data cycles.

## Introduction

Cardiovascular disease (CVD) continues to be the leading cause of mortality worldwide, with more than a 50% increase in CVD-related deaths from 1990 to 2019,^1^ and the increase in CVD is projected to accelerate over the next 5 years.^2^ In response, the American Heart Association recently developed the Predicting Risk of Cardiovascular Disease Events (PREVENT) equations to be used as a predictive risk assessment for CVD events and mortality.^3^ These equations address changes in risk factor prevalence across multiple populations, treatment and intervention approaches, and the rising burden of CVD subtypes.^3,4^ If used in clinically similar contexts to the American Heart Association/American College of Cardiology Pooled Cohort Equations (PCEs),^5^ the PREVENT equations have the potential to be a powerful means of communicating short-term and long-term risk of CVD.^3^

External validation is essential to aid the integration of the PREVENT equations into research and clinical practice. Therefore, we evaluated the calibration and discrimination of the PREVENT equations to estimate fatal CVD risk in an ongoing cohort study consisting of individuals representative of the overall US population, the National Health and Nutrition Examination Survey (NHANES), which is collected by the US National Center for Health Statistics.^6^ We then compared the performance of the PREVENT model against a current clinical standard, the PCE.^5^

## Methods

Data curation and analyses for this prognostic study took place from December 2023 through May 2024. Analytical approaches and reporting were performed consistent with the Transparent Reporting of a Multivariable Prediction Model for Individual Prognosis or Diagnosis (TRIPOD) reporting guidelines.^7^

### Study Cohort

Publicly available data were obtained from NHANES,^8^ which began in 1999 and continues to release public-use data in 2-year cycles. NHANES data collection is designed with stratified, multistage, and cluster sampling to create a randomized representative sample of the noninstitutionalized US general population.^9^ This investigation used data cycles from 1999 to 2010 to include the inception of the continuous NHANES study and ensure a 10-year follow-up period. The continuous NHANES study was reviewed and approved by the US National Center for Health Statistics Institutional Review Board. Participants in NHANES provided written informed consent.

### NHANES Data Collection and Extraction

NHANES variables used for the present investigation were based on risk factors from the PREVENT and PCE equations.^3,5^ Self-reported demographic information (age at the time of screening for the NHANES study, sex, and race) and medical history were collected via interview. Data on race and ethnicity are included in the current study for the purpose of comparing the present study cohort before and after accounting for the NHANES complex survey design, as well as comparing with the general US population. Physical examination measurements collected included anthropometric measurements and brachial blood pressure. Serum measurements of creatinine, glucose, total cholesterol (TC), and high-density lipoprotein (HDL) cholesterol were performed at a central laboratory. Estimated glomerular filtration rate (eGFR) was calculated using the Chronic Kidney Disease Epidemiology Collaboration 2021 creatinine equation.^10,11^ Hypertension was defined as either systolic blood pressure greater than or equal to 140 mm Hg, diastolic blood pressure greater than or equal to 90 mm Hg, physician diagnosis, or the use of antihypertensive medication.^12^ Diabetes was defined using the 2014 criteria established by the American Diabetes Association^13^: a fasting plasma glucose greater than or equal to 126 mg/dL (to convert to millimoles per liter, multiply by 0.0555) and/or use of insulin or antihyperglycemic medications. Chronic kidney disease was defined as an eGFR less than 60 mL/min/1.73 m^2^.^14^ Current or prior smoking status self-reported.

### Outcome Variables

The primary outcome of the present analyses was determined from *International Statistical Classification of Diseases and Related Health Problems, Tenth Revision (ICD-10)* codes as the combined cardiac (*ICD-10* codes I00-I09, I11, I13, and I20-I51) and cerebrovascular (*ICD-10* codes I60-I69) mortality, hereafter referred to as CVD mortality. It is important to note that these codes incorporate both atherosclerotic and nonatherosclerotic CVD mortality events without allowing for differentiation between CVD subtypes. All other mortality outcomes were collapsed into a single variable representing noncardiovascular mortality (hereafter referred to as competing risks). The use of National Death Index *ICD-10* codes for mortality outcomes in NHANES has been previously shown to match prevalence well^15^; however, there are discrepancies between claims data, self-reports, and aggregated data that should be considered. Data regarding mortality were obtained from the linked National Death Index database. The linked mortality information, which has been updated through December 31, 2019, is publicly available.^16^ Participants not matched with a death record were considered alive through the end of the follow-up period (December 31, 2019).

### Statistical Analysis

Continuous data are presented as mean and 95% CI, and categorical data are presented as percentage and 95% CI unless otherwise noted. Differences by PREVENT risk category were assessed with generalized linear regression models for continuous variables or generalized Poisson regression models for proportional variables. All analyses accounted for the NHANES complex survey design by adjusting for sampling weights (calculated according to NHANES guidelines), clustering, and stratification unless otherwise noted.

Present analyses focused on the base PREVENT model predicting composite fatal CVD. The base PREVENT model uses the following predictor variables: age at assessment, systolic blood pressure, HDL cholesterol, TC, eGFR, smoking status, use of antihypertensive medication, use of statin medication, and diagnosis of diabetes. These variables were used to calculate the 10-year risk of CVD based on the sex-specific PREVENT equations (eTable 1 in Supplement 1).^3^

Per recommendations of the PREVENT working group, the PREVENT risk scores were entered into hazard regression models accounting for the competing risk of noncardiovascular mortality using the methods of Fine and Gray^17,18^ and adjusted for sample weights and clustering. The time-to-event was determined as years of follow-up from their NHANES examination date. The rationale for using this timescale instead of an age-based scale (used by the PREVENT working group^3^) was that this allowed for validation of the 10-year risk predicted by the PREVENT equations in a cohort with adequate follow-up duration. Subdistribution hazard ratios (HRs) were assessed per 1%-increase in predicted risk, and model discrimination was assessed with the Harrell C statistic^19^; a C statistic between 0.70 and 0.80 is considered good, and greater than or equal to 0.80 is excellent.^20^ The 95% CIs for the C statistic were created using a jackknife approach.^21^

Predictive analyses on the PREVENT competing-risks model were made with the approach described previously by Zheng et al.^22,23^ In brief, competing risks (cardiovascular and noncardiovascular mortality) receiver-operator characteristic (ROC) and 95% CIs were generated at the 10-year mark. This time point was chosen to be consistent with previous risk assessments^5^ and American Heart Association recommendations.^24^ Unweighted competing-risk ROC curves were created by bootstrapping with 500 repetitions. Sensitivity analyses were conducted using conventional ROC adjusted for survey sample weights, clustering, and stratification.

Calibration of the PREVENT equation with the NHANES dataset was assessed as the slope of predicted vs observed risk of CVD mortality.^25,26,27^ The graphical relationship was modeled using restricted cubic spline regression with 3 knots located at the 10th, 50th, and 90th percentiles.^28^ The slope of this relationship is a measure of calibration, such that a slope of 1.0 indicates perfect calibration. Slopes less than 1.0 indicate an underestimated risk, whereas slopes greater than 1.0 indicate an overestimated risk or an underfit model.^29^

Comparisons between the PREVENT equations and the PCE were made using the discrimination and calibration statistics. Similar to our approach for the PREVENT risk scores, a Fine and Gray competing risk regression model^17,18^ was created for the PCE estimated risk. Discriminatory ability was determined for both the PCE and PREVENT models with Harrell C statistic and jackknife CI.^19,21^ Comparisons of discrimination between models were conducted with log-likelihood ratios. Calibration in the PCE model was assessed as the slope of predicted vs observed risk of CVD mortality.^25,26,27^

The categorical net reclassification index (NRI) was used to compare the PCE and PREVENT equations in the original unweighted cohort according to the methods of Pencina et al.^30^ Participants were stratified by PCE or PREVENT risk predicted as low (<5.0%), low-moderate (5.0% to <7.5%), moderate-high (7.5% to <10.0%), or high (≥10.0%) risk, in accordance with previous studies.^24,25,31^ The NRI analysis was performed specifically at the 10-year mark,^24^ and models were run with 500 bootstrap replications.

Following the development methods for the PREVENT equations, we performed sensitivity analyses by removing participants with extreme values of clinical variables (using the following cutoffs from the PREVENT working group^3^: body mass index [calculated as weight in kilograms divided by height in meters squared] <18.5 or ≥40.0, systolic blood pressure <90 or >200 mm Hg, TC <130 or >320 mg/dL [to convert to millimoles per liter, multiply by 0.0259], and HDL cholesterol <20 or >100 mg/dL [to convert to millimoles per liter, multiply by 0.0259]). Furthermore, we performed sensitivity analyses altering the definition of the smoking status variable in PREVENT calculations from current/previous smoker to only current smoker, to account for differences in the PREVENT cohorts’ smoking definitions.

All statistical methods were run in Stata statistical software version 18.0 (StataCorp) or R statistical software version 4.1.2 (R Project for Statistical Computing). Competing-risks models were run in Stata, with postestimation of Harrell C statistic using the user-created somersd package.^32^ Calibration, competing-risks ROC, and NRI analyses were performed in Stata and R using the CalibrationCurves,^33^ survCompetingRisk,^22,23^ and nricens^34^ packages. All statistical tests were evaluated for significance at a nominal type I error rate of α = .05 with 2-sided *P* < .05.

## Results

From the 62 160 individuals who participated in NHANES between 1999 and 2010, exclusion for missing data (risk score variables or outcomes) or prevalent atherosclerotic CVD (ie, chronic heart failure, myocardial infarction, coronary artery disease, or stroke)^3,5^ resulted in a final study dataset consisting of 24 582 participant records representing 172.9 million US adults (mean age, 45.0 years [95% CI, 44.6-45.4 years]; 52.1% women [95% CI, 51.5%-52.6%]) after adjustment for the complex survey design of NHANES with sample weights, clustering, and stratification.^9^ Demographic characteristics stratified by PREVENT risk score are presented in Table 1; characteristics separated by sex are presented in eTable 2 in Supplement 1, and a comparison with the PREVENT cohorts is presented in eTable 3 in Supplement 1. During the follow-up period (13.4 years; range, 0.1-20.8 years) in the unweighted cohort, a total of 4240 deaths (17.3%) occurred, with 1195 CVD deaths (4.9% of total cohort) and 3045 non-CVD deaths (12.4%). The majority of non-CVD deaths were due to cancers (4.1%), followed by chronic respiratory disease (1.0%) and Alzheimer disease (0.8%); 1878 of the unweighted cohort participants (7.6%) in the present cohort were cancer survivors.

**Table 1.** Cohort Demographics Stratified by 10-Year CVD Risk Score

Characteristic	Participants, mean (95% CI), %				*P* value
	<5% 10-y CVD risk	5% to <7.5% 10-y CVD risk	7.5% to <10% 10-y CVD risk	≥10% 10-y CVD risk	
Original cohort and survey-adjusted cohort characteristics					
Sample size, unweighted No.	14 677	1804	1261	6840	NA
Sample size, weighted No., millions (95% CI)	33.1 (31.8-34.5)	117.1 (115.2-119.0)	14.0 (13.2-14.9)	8.7 (8.0-9.4)	NA
Sample-weighted measurements					
Age, mean (95% CI), y	36.6 (36.3-36.9)	53.4 (53.0-53.8)	57.2 (56.5-57.8)	67.9 (67.5-68.2)	<.001
Sex					
Female	53.7 (52.9-54.5)	45.5 (42.8-48.3)	47.6 (44.3-50.9)	50.4 (48.8-51.9)	.006
Male	46.3 (45.5-47.1)	54.5 (51.7-57.2)	52.4 (49.1-55.7)	49.6 (48.1-51.2)	
Race and ethnicity^a^					
Mexican-American	9.7 (8.4-11.2)	5.6 (4.4-7.1)	5.3 (4.1-6.8)	4.7 (3.5-6.2)	.41
Non-Hispanic Black	10.8 (8.0-11.2)	8.9 (7.4-10.6)	8.6 (6.9-10.6)	9.5 (8.0-11.2)	
Non-Hispanic White	67.9 (65.3-70.3)	77.4 (74.1-80.3)	76.3 (72.3-79.8)	78.3 (75.4-80.9)	
Other Hispanic	5.8 (4.6-7.2)	4.2 (2.9-4.5)	5.1 (3.4-7.5)	3.9 (2.7-5.6)	
Other or multiracial	5.9 (5.1-6.8)	4.0 (2.9-5.6)	4.8 (3.4-6.6)	3.7 (3.0-4.5)	
Body mass index, mean (95% CI)^b^	27.6 (27.4-27.8)	29.4 (29.0-29.7)	29.9 (29.4-30.4)	29.3 (29.1-29.5)	.17
Systolic blood pressure, mean (95% CI), mm Hg	115.8 (115.4-116.1)	126.4 (125.6-127.2)	130.4 (129.2-131.6)	138.8 (138.0-139.7)	<.001
Diastolic blood pressure, mean (95% CI), mm Hg	70.8 (70.5-71.2)	75.4 (74.5-76.4)	75.4 (74.4-76.4)	69.4 (68.8-70.1)	<.001
Antihypertension medication	6.1 (5.6-6.7)	25.7 (23.3-28.2)	32.9 (29.6-36.3)	55.9 (54.1-57.7)	<.001
Total cholesterol, mean (95% CI), mg/dL	196.0 (195.2-196.8)	216.5 (213.8-219.2)	216.6 (213.1-220.0)	207.2 (205.4-209.0)	<.001
HDL cholesterol, mean (95% CI), mg/dL	53.6 (53.2-54.0)	50.9 (50.0-51.9)	51.8 (50.4-53.2)	52.0 (51.4-52.7)	.29
Statin use	2.2 (1.8-2.6)	11.1 (9.5-13.0)	14.7 (12.0-17.9)	25.5 (24.3-26.8)	<.001
Non-HDL cholesterol, mean (95% CI), mg/dL^c^	142.4 (141.5-143.2)	165.6 (162.9-168.3)	164.7 (161.2-168.2)	155.2 (153.3-157.0)	<.001
Serum glucose, mean (95% CI), mg/dL^d^	89.2 (88.9-89.6)	99.6 (97.8-101.5)	104.0 (101.3-106.7)	113.2 (111.7-114.7)	<.001
Antidiabetes medication	0.7 (0.6-0.9)	4.9 (3.8-6.2)	7.0 (5.3-9.2)	18.1 (17.0-19.3)	<.001
Estimated glomerular filtration rate, mean (95% CI), mL/min/1.73 m^2^	105.5 (105.0-106.0)	93.0 (92.0-93.9)	89.8 (88.6-91.0)	78.7 (77.9-79.5)	<.001
Current or former smokers	41.7 (40.1-43.2)	59.1 (56.3-61.8)	58.3 (54.7-61.9)	59.5 (57.6-61.4)	.32
Hypertension	18.2 (17.3-19.2)	49.8 (47.2-52.3)	58.5 (54.8-62.1)	81.4 (80.2-82.6)	<.001
Diabetes	1.7 (1.5-2.0)	10.9 (9.3-12.7)	16.1 (13.4-19.3)	31.7 (30.2-33.4)	<.001
Chronic kidney disease	0.4 (0.3-0.6)	1.4 (0.9-2.3)	3.3 (2.2-5.0)	17.0 (15.8-18.2)	<.001

Abbreviations: CVD, cardiovascular disease; HDL, high-density lipoprotein; NA, not applicable.

SI conversion factors: To convert glucose to millimoles per liter, multiply by 0.0555; to convert HDL cholesterol to millimoles per liter, multiply by 0.0259; to convert total cholesterol to millimoles per liter, multiply by 0.0259.

^a^ Other Hispanic included any self-reported Hispanic individuals who were not Mexican-American. The other/multiracial category is not detailed further in the National Health and Nutrition Examination Survey methods.

^b^ Body mass index is calculated as weight in kilograms divided by height in meters squared. A total of 312 original values were missing.

^c^ Non-HDL cholesterol is calculated as total cholesterol minus HDL cholesterol.

^d^ One original value was missing.

### PREVENT Risk Score Performance

**Figure 1.**
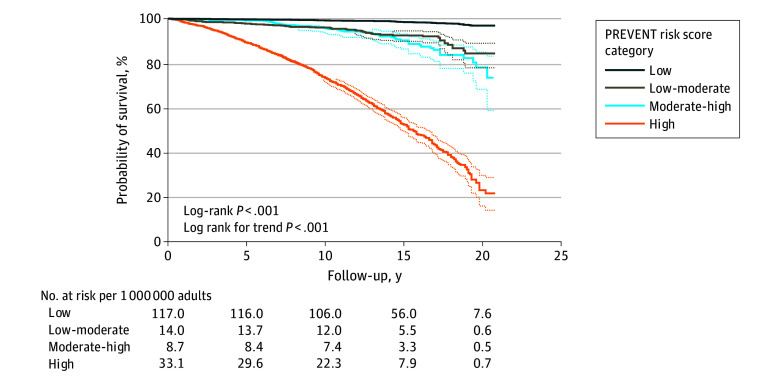
Kaplan-Meier Survival Curve Stratified by Predicting Risk of Cardiovascular Disease Events (PREVENT) Risk Score Category The risk score categories are defined as low (<5.0%), low-moderate (5.0% to <7.5%), moderate-high (7.5% to <10.0%), and high (≥10.0%) risk. Dotted lines denote 95% CIs.

Kaplan-Meier analysis revealed significant differences in CVD-related survival across the PREVENT risk strata (Figure 1). In univariable competing-risks regression, a 1% increase in PREVENT risk was significantly associated with increased CVD mortality (HR, 1.090; 95% CI, 1.087-1.094). Discrimination was classified as excellent (C statistic, 0.890; jackknife 95% CI, 0.881-0.898). These findings remained consistent when analyzed by sex. For men, PREVENT risk was similarly associated with CVD mortality risk (HR, 1.089; 95% CI, 1.081-1.098), and the C statistic was classified as excellent (0.873; jackknife 95% CI, 0.860-0.886). Results were similar for women (HR, 1.090; 95% CI, 1.080-1.100), albeit with a numerically greater C statistic (0.904; jackknife 95% CI, 0.892-0.916).

**Figure 2.**
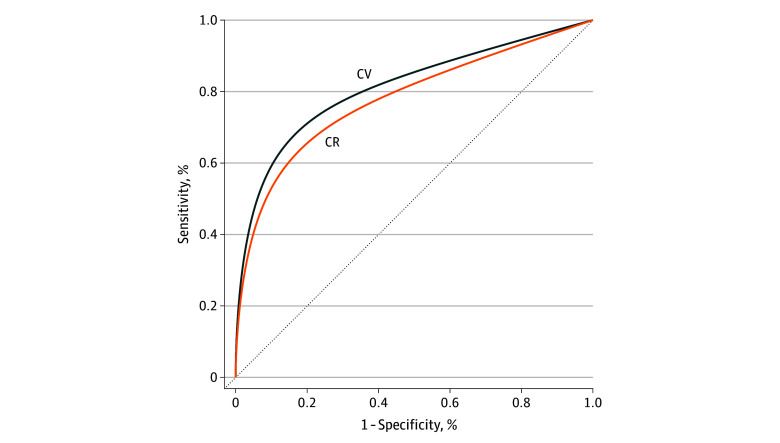
Receiver-Operator Characteristic Curves Demonstrating Sensitivity and Specificity of Predicting Risk of Cardiovascular Disease Events Score for Cardiovascular (CV) and Competing Risk (CR) Mortality in the Overall Cohort Dotted line denotes the expected performance of a perfectly random classifier.

Competing-risks ROC analyses supported the above results. In the overall cohort, area under the curve (AUC) for CVD mortality was 0.813 (95% CI, 0.810-0.816) (Figure 2). For men, the AUC for CVD mortality was 0.828 (95% CI, 0.824-0.832) (eFigure 1 in Supplement 1), whereas for women, the AUC for CVD mortality was 0.804 (95% CI, 0.797-0.808) (eFigures 2 and 3 in Supplement 1).

**Figure 3.**
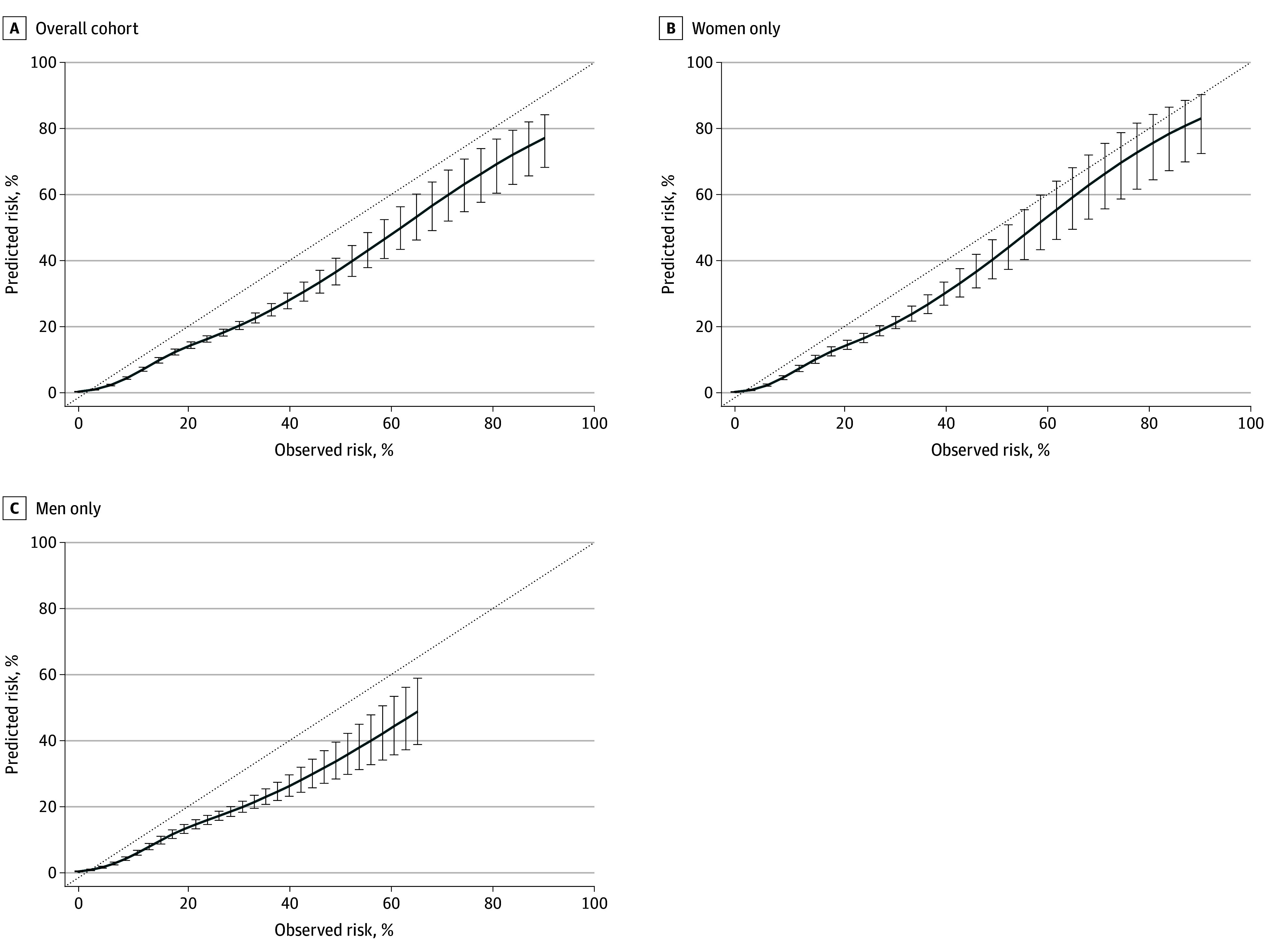
Calibration Assessed as the Slope of the Observed vs Predicted Risk Percentages According to Predicting Risk of Cardiovascular Disease Events Score Solid lines and error bars denote the mean line of regression and 95% CIs. Dotted line denotes perfect calibration (ie, a slope of 1.0).

Observed and predicted event rates for CVD mortality are presented in Figure 3 for visual assessment of calibration. In the overall cohort, modest underfitting of the model was indicated by the slope (1.13; 95% CI, 1.06-1.21). Analyses stratified by sex indicated this was similarly observed for women (slope, 1.17; 95% CI, 1.06-1.28), but suggested adequate calibration for men (slope, 1.09; 95% CI, 0.99-1.19).

### Comparison Between PREVENT and PCE Risk Predictions

In univariable competing-risks regression, an increase in PCE risk was associated with a greater risk of CVD mortality (HR, 1.050; 95% CI, 1.049-1.054). Although the C statistic was classified as excellent (0.880; jackknife 95% CI, 0.868-0.886), PCE discrimination was numerically lower than the PREVENT model in the overall cohort, demonstrated by the lack of overlap in 95% CIs. Likelihood tests confirmed a statistically significantly improved discrimination by PREVENT compared with PCE (χ^2^ = 113.8; *P* < .001). In the overall cohort and in contrast to PREVENT assessments, the PCE demonstrated an overfitting of the model (slope, 0.77; 95% CI, 0.73-0.81).

Reclassification from PCE to PREVENT risk score is shown in Table 2. The overall NRI indicated significant improvement when using PREVENT instead of PCE (NRI, 0.093; 95% CI, 0.073 to 0.115). This was primarily the result of downward reclassification of individuals for whom risk may have been overestimated by PCE (downward NRI, 0.139; 95% CI, 0.134 to 0.145). Reclassification resulted in sex-specific improvement of risk estimation for men (NRI, 0.212; 95% CI, 0.182 to 0.243) but not for women (NRI, −0.003; 95% CI, −0.029 to 0.025). Reclassification tables by sex are detailed in eTables 4 and 5 in Supplement 1.

**Table 2.** Reclassification Table for the Overall Cohort, Created Alongside Net Reclassification Index Analyses^a^

10-y Risk estimated by PCE	10-y Risk estimated by PREVENT				Total counts^b^
	Low risk (<5.0%)	Low-moderate risk (5.0% to <7.5%)	Moderate-high risk (7.5% to <10.0%)	High risk (≥10.0%)	
Participants who experienced a CVD mortality event					
Low risk (<5.0%)	26 (81.3)	4 (12.5)	2 (6.3)	0	32
Low-moderate risk (5.0% to <7.5%)	5 (35.7)	6 (42.9)	2 (14.3)	1 (7.1)	14
Moderate-high risk (7.5% to <10.0%)	7 (35.0)	5 (25.0)	3 (15.0)	5 (25.0)	20
High risk (≥10.0%)	5 (0.8)	13 (2.0)	13 (2.0)	632 (95.3)	663
Total counts	43	28	20	638	729
Participants who did not experience a CVD mortality event					
Low risk (<5.0%)	10 676 (96.5)	316 (2.9)	45 (0.4)	23 (0.2)	11 060
Low-moderate risk (5.0% to <7.5%)	924 (61.8)	323 (21.6)	168 (11.2)	79 (5.3)	1494
Moderate-high risk (7.5% to <10.0%)	604 (53.8)	156 (13.9)	199 (17.7)	164 (14.6)	1123
High risk (≥10.0%)	686 (11.5)	688 (11.5)	622 (10.4)	3994 (66.7)	5990
Total counts	12 890	1483	1034	4260	19 667

Abbreviations: PCE, Pooled Cohort Equations; PREVENT, Predicting Risk of Cardiovascular Disease Events.

^a^ Entries in each cell are given as count (percentage, %) where the percentages are calculated as the cell count over the row sum.

^b^ Note that the total counts do not sum to the total number of participants in the cohort; this is because any participants who did not experience an event but were censored before the time point of interest (10 years) are not included in analysis. In addition, any participants who experienced an event, but after the 10-year mark, are included in the counts for those who did not experience an event before 10 years.

### Sensitivity Analyses

In the total cohort, extreme values were observed for 1672 participants (6.8%) for body mass index, 224 participants (0.9%) for systolic blood pressure, 821 participants (3.3%) for TC, and 302 participants (1.2%) for HDL cholesterol. The demographic characteristics of this subcohort, overall and by sex, are presented in eTable 6 in Supplement 1. The results were not substantially different from those of the primary analyses (eAppendix in Supplement 1). In addition, analyses performed with a strict definition of current smokers did not substantially alter present results (data not shown).

Conventional ROC analyses were performed after adjusting for survey sample weights and clustering. The results were not substantially different from those of the primary analyses (eAppendix in Supplement 1).

## Discussion

In this prognostic study of a US-based cohort, the novel American Heart Association PREVENT equations demonstrated strong capabilities to predict CVD mortality over a 10-year follow-up. The PREVENT risk scores demonstrated excellent discrimination of CVD mortality risk in the overall cohort. Despite moderate discrepancies observed in the calibration assessments, the C statistics consistently supported good-to-excellent discrimination in the present results. In the overall cohort and with the removal of extreme clinical variable values, the C statistics were similar to, or greater than, those observed by the PREVENT working group. These findings support the incorporation of the PREVENT equations in clinical settings to inform patient-clinician discussions on CVD risk and appropriateness of intervention. Furthermore, sex-specific improvements in CVD risk estimation were noted in the NRI analyses, suggesting that the PREVENT equations may better capture sex-based discrepancies in the incidence and impact of cardiometabolic disease.

CVD risk estimation tools used in clinical practice (eg, PCE, QRISK2,^35^ or SCORE^36^) are valid if they demonstrate sufficient calibration and discrimination, such that predicted risk estimates are accurate and informative.^25,37,38^ In the development of the PREVENT equations, discrimination (range of median C statistics across cohorts, 0.745-0.794) and calibration (median slope for all CVD was 0.94 in men and 1.03 in women) were good for both men and women.^3^ In the present study using NHANES data, the observed discrimination was significantly higher than the C statistics reported by the American Heart Association working group, supporting the PREVENT model’s discriminatory ability. However, calibration in the present overall cohort suggested the model may be underfit. This indicates that each 1% increase in the PREVENT model was associated with an increase in observed risk greater than 1% (ie, underprediction). This contrasts with the PCE model, which was overfit, indicating a degree of overprediction of risk.^29^

There are several possible reasons for this modest discrepancy between the calibration and discrimination of the PREVENT equations in the current study and those reported in the original publication of the PREVENT equations.^3^ First, we did not initially exclude participants with extreme values of systolic blood pressure, TC, HDL cholesterol, or body mass index as was done in the PREVENT report.^3^ However, exclusion of these participants in sensitivity analyses did not substantially affect calibration or discrimination.

A second potential reason for our findings is subtle differences in the present cohort compared with the PREVENT development and validation cohorts. Although most traditional risk factors were similar, factors of note are the modestly lower age of the current cohort, differences in racial and ethnic backgrounds, and smoking status. The PREVENT cohorts were composed of participants aged around 52 years old,^3^ compared with approximately 45 years in the present cohort. Although the present study followed the recommendations of the PREVENT working group and did not analyze race,^39^ there remains the possibility of differences in social determinants of health that may have impacted our findings.^40,41^ In addition, the present analyses included current and former smokers in smoking status owing to variable definitions across cohorts used in the PREVENT equation development.^3^ However, analyses performed with a strict definition of current smokers did not substantially alter present results. Furthermore, chronic conditions not explicitly discussed may have differed between the present cohort and the PREVENT cohorts. For example, 1878 of the unweighted cohort participants (7.6%) in the present cohort were cancer survivors; it is well-established that prior anticancer treatments lead to accelerated vascular aging,^42,43^ resulting in greater risk of CVD.^44,45,46^ Other chronic diseases of aging, such as cerebral small vessel disease^47^ or coronary artery calcification,^48^ are not measured in many cohort studies because of technical and financial limitations but may have a considerable impact on the risk of CVD as well. The NHANES database does not contain this information, but future studies should investigate the value of incorporating chronic conditions into PREVENT risk estimation.

A third possibility relates to the determination of outcomes in the present study. The NHANES database is linked to the National Death Index, which allows for adjudication of mortality outcomes on the basis of *ICD-10* codes. However, this limits the identification of nonfatal CVD events.^49^ It would be expected that including active surveillance and ascertainment of nonfatal events would correct an overestimation of risk, and potentially refine an underfit model.^25^ Importantly, the use of *ICD-10* codes also reduces identification of subtypes of CVD; for example, the *ICD-10* codes for cerebrovascular disease (I60-I69) include both atherosclerotic CVD (ischemic stroke) and nonatherosclerotic CVD (intracerebral hemorrhage). This is an important consideration of the present findings and is an inherent limitation in the NHANES dataset.

### Limitations

Limitations of the present study include those discussed previously, primarily that NHANES does not include surveillance of participants, preventing the incorporation of nonfatal CVD events. Furthermore, it was not possible to fully account for the complex survey design of the NHANES study in some of the more recently developed statistical approaches used in the present study, namely competing-risk ROC curves and the categorical NRI. Although sensitivity analyses were used in the present study to mitigate these statistical limitations, further studies in other large cohorts with longitudinal surveillance to address the first limitation are warranted.

## Conclusions

The PREVENT equations demonstrated good-to-excellent discrimination in the present US cohort extracted from the NHANES database. Taken in sum, the present findings provide evidence in support of the validity of the PREVENT equations for application in the intended target population.

## Notes

Supplement 1. **eAppendix. **Sensitivity analyses results **eTable 1.** The base PREVENT 10-year risk estimation model equations, separated by sex **eTable 2.** Overall cohort demographics separated by sex **eTable 3.** Sex-stratified survey-weighted cohort demographics compared to the development and validation cohorts in PREVENT **eTable 4.** Reclassification table for only males from the overall cohort, created alongside net reclassification index analyses **eTable 5.** Reclassification table for only females from the overall cohort, created alongside net reclassification index analyses **eTable 6.** Demographic characteristics of the sub-cohort consisting of participants who did not have ‘extreme’ clinical variable values as described in the Methods **eFigure 1.** Competing-risks receiver-operator characteristic curves demonstrating PREVENT sensitivity and specificity for cardiovascular and non-cardiovascular mortality specifically in male participants **eFigure 2.** Competing-risks receiver-operator characteristic curves demonstrating PREVENT sensitivity and specificity for cardiovascular and non-cardiovascular mortality specifically in female participants **eFigure 3.** Competing-risks receiver-operator characteristic curves demonstrating PREVENT sensitivity and specificity for cardiovascular and non-cardiovascular mortality specifically after excluding participants who had ‘extreme’ clinical variable values

Supplement 2.
Data Sharing Statement
