# Supplementary material for: External Validation of the American Heart Association PREVENT Cardiovascular Disease Risk Equations
Source: JAMA Netw Open. 2024 Oct 11;7(10):e2438311. doi: 10.1001/jamanetworkopen.2024.38311 (PMC11470385; doi:10.1001/jamanetworkopen.2024.38311)
Supplement: Supplement 1. — eAppendix. Sensitivity analyses results eTable 1. The base PREVENT 10-year risk estimation model equations, separated by sex eTable 2. Overall cohort demographics separated by sex eTable 3. Sex-stratified survey-weighted cohort demographics compared to the development and validation cohorts in PREVENT eTable 4. Reclassification table for only males from the overall cohort, created alongside net reclassification index analyses eTable 5. Reclassification table for only females from the overall cohort, created alongside net reclassification index analyses eTable 6. Demographic characteristics of the sub-cohort consisting of participants who did not have ‘extreme’ clinical variable values as described in the Methods eFigure 1. Competing-risks receiver-operator characteristic curves demonstrating PREVENT sensitivity and specificity for cardiovascular and non-cardiovascular mortality specifically in male participants eFigure 2. Competing-risks receiver-operator characteristic curves demonstrating PREVENT sensitivity and specificity for cardiovascular and non-cardiovascular mortality specifically in female participants eFigure 3. Competing-risks receiver-operator characteristic curves demonstrating PREVENT sensitivity and specificity for cardiovascular and non-cardiovascular mortality specifically after excluding participants who had ‘extreme’ clinical variable values [file jamanetwopen-e2438311-s001.pdf]

# Supplemental Online Content

Scheuermann B, Brown A, Colburn T, Hakeem H, Chow CH, Ade C. External validation of the American Heart Association PREVENT Cardiovascular Disease Risk Equations. *JAMA Netw Open*. 2024;7(10):e2438311. doi:10.1001/jamanetworkopen.2024.38311

**eAppendix.** Sensitivity analyses results

**eTable 1.** The base PREVENT 10-year risk estimation model equations, separated by sex

**eTable 2.** Overall cohort demographics separated by sex

**eTable 3.** Sex-stratified survey-weighted cohort demographics compared to the development and validation cohorts in PREVENT

**eTable 4.** Reclassification table for only males from the overall cohort, created alongside net reclassification index analyses

**eTable 5.** Reclassification table for only females from the overall cohort, created alongside net reclassification index analyses

**eTable 6.** Demographic characteristics of the sub-cohort consisting of participants who did not have ‘extreme’ clinical variable values as described in the Methods

**eFigure 1.** Competing-risks receiver-operator characteristic curves demonstrating PREVENT sensitivity and specificity for cardiovascular and non-cardiovascular mortality specifically in male participants

**eFigure 2.** Competing-risks receiver-operator characteristic curves demonstrating PREVENT sensitivity and specificity for cardiovascular and non-cardiovascular mortality specifically in female participants

**eFigure 3.** Competing-risks receiver-operator characteristic curves demonstrating PREVENT sensitivity and specificity for cardiovascular and non-cardiovascular mortality specifically after excluding participants who had ‘extreme’ clinical variable values

This supplemental material has been provided by the authors to give readers additional information about their work.

© 2024 Scheuermann B et al. *JAMA Network Open*.

**eAppendix. Sensitivity analyses results**

Sensitivity Analyses – Extreme Clinical Values

In univariate competing-risks regression, each 1% increase in PREVENT risk score significantly increased risk of CVD mortality (HR 1.09, 95% CI: 1.087-1.099). Discrimination was classified as excellent (c-statistic 0.893, jackknife 95% CI: 0.884-0.902). Hazard regression results and discrimination in the sub-cohort were similar for men and women (data not shown). In competing-risks ROC curve analysis with the overall sub-cohort (Supplementary Figure e3), PREVENT risk predicted CVD mortality with an AUC of 0.819 (95% CI: 0.816-0.822) and non-CVD mortality with an AUC of 0.79 (95% CI: 0.786-0.792). The calibration slope in the sensitivity cohort was 1.17 (95% CI: 1.08-1.25). In only men without extreme clinical values, the calibration slope was 1.10 (95% CI: 0.98-1.22) while in women it was 1.24 (95% CI: 1.11-1.36).

Sensitivity Analyses – Conventional ROC

Conventional ROC were performed with adjustments for NHANES survey sample weights and clustering. In the overall cohort, the AUC was 0.86 (95% CI: 0.844-0.870, $p<0.01$). In only men, the AUC was 0.83 (95% CI: 0.828-0.839, $p<0.01$). In only women, the AUC was 0.88 (95% CI: 0.857-0.900, $p<0.01$).

© 2024 Scheuermann B et al. *JAMA Network Open*.

**eTable 1.** The base PREVENT 10-year risk estimation model equations, separated by sex.

| **Composite CVD outcomes, 10-year risk estimation** | |
|---|---|
| **Men** | **log-Odds** = -3.031168 + 0.7688528 × (age – 55) /10 + 0.0736174 × ((TC – HDL) × 0.02586 – 3.5) – 0.0954431 × (HDL × 0.02586 – 1.3) /0.3 – 0.4347345 × (min(SBP, 110) – 110) /20 + 0.3362658 × (max(SBP, 110) – 130) /20 + 0.7692857 × (if diabetes) + 0.4386871 × (if current smoker) + 0.5378979 × (min(eGFR, 60) – 60) / -15 + 0.0164827 × (max(eGFR, 60) – 90) / -15 + 0.288879 × (if using anti-hypertensive medication) – 0.1337349 × (if using statin) – 0.0475924 × (if using anti-hypertensive medication) × (max(SBP, 110) – 130) /20 + 0.150273 × (if using statin) × ((TC – HDL) × 0.02586 – 3.5) – 0.0517874 × (age – 55) /10 × ((TC – HDL) × 0.02586 – 3.5) + 0.0191169 × (age – 55) /10 × (HDL × 0.02586 – 1.3) /0.3 – 0.1049477 × (age – 55) /10 × (max(SBP, 110) – 130) /20 – 0.2251948 × (age – 55) /10 × (if diabetes) – 0.0895067 × (age – 55) /10 × (if current smoker) – 0.1543702 × (age – 55) /10 × (min(eGFR, 60) – 60) / -15 |
| **Women** | **log-Odds** = -3.307728 + 0.7939329 × (age – 55) /10 + 0.0305239 × ((TC – HDL) × 0.02586 – 3.5) – 0.1606857 × (HDL × 0.02586 – 1.3) /0.3 – 0.2394003 × (min(SBP, 110) – 110) /20 + 0.360078 × (max(SBP, 110) – 130) /20 + 0.8667604 × (if diabetes) + 0.5360739 × (if current smoker) + 0.6045917 × (min(eGFR, 60) – 60) / -15 + 0.0433769 × (max(eGFR, 60) – 90) / -15 + 0.3151672 × (if using anti-hypertensive medication) – 0.1477655 × (if using statin) – 0.0663612 × (if using anti-hypertensive medication) × (max(SBP, 110) – 130) /20 + 0.1197879 × (if using statin) × ((TC – HDL) × 0.02586 – 3.5) – 0.0819715 × (age – 55) /10 × ((TC – HDL) × 0.02586 – 3.5) + 0.0306769 × (age – 55) /10 × (HDL × 0.02586 × 0.02586 – 1.3) /0.3 – 0.0946348 × (age – 55) /10 × (max(SBP, 110) – 130) /20 – 0.27057 × (age – 55) /10 × (if diabetes) – 0.078715 × (age – 55) /10 × (if current smoker) – 0.1637806 × (age – 55) /10 × (min(eGFR, 60) – 60) / -15 |

© 2024 Scheuermann B et al. *JAMA Network Open*.

**eTable 2.** Overall cohort demographics separated by sex[a].

| | Overall Cohort | Males | Females |
|---|---|---|---|
| **Sample Size, Unweighted n** | 24582 | 11597 | 12985 |
| **Sample Size, Weighted n (in millions)** | 172.9 | 82.8 (81.9-83.8) | 90.0 (89.0-91.0) |
| **Age, years** | 45.0 (44.6-45.4) | 43.9 (43.5-44.4) | 46.0 (45.5-46.4) |
| **Race/Ethnicity, %** [b] | | | |
| **Mexican-American** | 8.2 (7.0-9.6) | 9.0 (7.7-10.6) | 7.4 (6.3-8.8) |
| **Non-Hispanic Black** | 10.3 (9.0-11.6) | 9.6 (8.5-10.8) | 10.8 (9.4-12.4) |
| **Non-Hispanic White** | 71.0 (68.5-73.5) | 71.1 (68.6-73.4) | 71.0 (68.3-73.6) |
| **Other Hispanic** | 5.2 (4.1-6.7) | 5.1 (4.0-6.6) | 5.3 (4.1-6.9) |
| **Other/Multi-Racial** | 5.2 (4.6-6.0) | 5.1 (4.4-6.0) | 5.3 (4.6-6.2) |
| **BMI, kg/m$^2$** [c] | 28.2 (28.0-28.3) | 28.2 (28.0-28.3) | 28.2 (28.0-28.4) |
| **SBP, mmHg** | 121.8 (121.4-122.2) | 123.3 (122.9-123.8) | 120.4 (119.8-120.9) |
| **DBP, mmHg** | 71.1 (70.8-71.5) | 72.9 (72.6-73.3) | 69.5 (69.1-69.9) |
| **Anti-HTN Medication, %** | 18.6 (17.7-19.4) | 16.7 (15.7-17.7) | 20.3 (19.3-21.4) |
| **Total Cholesterol, mg/dL** | 200.8 (199.9-201.7) | 199.7 (198.6-200.8) | 201.8 (200.7-202.9) |
| **HDL, mg/dL** | 53.0 (52.6-53.4) | 47.3 (46.9-47.7) | 58.2 (57.7-58.8) |
| **Non-HDL-c, mg/dL** | 147.8 (146.9-148.7) | 152.4 (151.3-153.5) | 143.6 (142.4-144.8) |
| **Statin Use, %** | 8.0 (7.5-8.6) | 8.5 (7.7-9.3) | 7.6 (6.9-8.3) |
| **Serum Glucose, mg/dL**[d] | 95.4 (94.9-95.9) | 97.3 (96.7-98.0) | 93.6 (93.0-94.2) |
| **Anti-DM Medication, %** | 4.7 (4.4-5.1) | 4.7 (4.3-5.1) | 4.8 (4.3-5.2) |
| **eGFR, mL/min per 1.73 m$^2$** | 98.6 (98.0-99.2) | 99.3 (98.7-99.9) | 97.9 (97.2-98.6) |
| **Current/Former Smokers, %** | 47.3 (46.0-48.6) | 54.0 (52.3-55.6) | 41.2 (39.8-42.6) |
| **Hypertension, %** | 34.9 (33.8-36.0) | 34.6 (33.3-36.0) | 35.2 (33.9-36.5) |
| **Diabetes Mellitus, %** | 8.9 (8.5-9.5) | 9.1 (8.4-9.8) | 8.8 (8.3-9.5) |
| **CKD, %** | 3.8 (3.5-4.1) | 2.6 (2.3-2.9) | 4.9 (4.4-5.4) |

© 2024 Scheuermann B et al. *JAMA Network Open*.

[a] All values are presented as mean and 95% confidence interval. BMI: body-mass index; SBP: systolic blood pressure; DBP: diastolic blood pressure; HTN: hypertension; HDL: high-density lipoprotein; DM: diabetes mellitus; eGFR: estimated glomerular filtration rate; CKD: Chronic Kidney Disease.

[b] Other Hispanic included any self-reported Hispanic individuals who were not Mexican-American. The Other/Multi-racial category is not detailed further in NHANES methods.

[c] n=312 original values missing.

[d] n=1 original value missing.

© 2024 Scheuermann B et al. *JAMA Network Open*.

**eTable 3.** Sex-stratified survey-weighted cohort demographics compared to the development and validation cohorts in PREVENT[a].

| | Present Study (Males) | Present Study (Females) | P.D. (Males) | P.D. (Females) | P.V. (Males) | P.V. (Females) |
|---|---|---|---|---|---|---|
| **Sample Size, Weighted n (in millions)** | 82.8 (81.9-83.8) | 90.0 (89.0-91.0) | 1442091 | 1839828 | 1435203 | 1894882 |
| **Age, years** [b] | 43.9 (43.5-44.4) | 43.9 (43.5-44.4) | 52 (12) | 53 (13) | 52 (12) | 52 (13) |
| **Black, %** [c] | 9.6 (8.5-10.8) | 9.6 (8.5-10.8) | (8.0) | (10) | (8.2) | (10) |
| **BMI, kg/m$^2$** [b] | 28.2 (28.0-28.3) | 28.2 (28.0-28.3) | 29 (4) | 29 (5) | 29 (4) | 28 (5) |
| **SBP, mmHg** | 123.3 (122.9-123.8) | 123.3 (122.9-123.8) | 127 (15) | 123 (16) | 128 (15) | 123 (16) |
| **DBP, mmHg** | 72.9 (72.6-73.3) | 72.9 (72.6-73.3) | --- | --- | --- | --- |
| **Anti-HTN Medication, %** [c] | 16.7 (15.7-17.7) | 16.7 (15.7-17.7) | (27) | (23) | (29) | (24) |
| **Total Cholesterol, mg/dL** [d] | 199.7 (198.6-200.8) | 199.7 (198.6-200.8) | 189.5 (30.9) | 193.4 (30.9) | 189.5 (30.9) | 193.4 (30.9) |
| **HDL, mg/dL** [d] | 47.3 (46.9-47.7) | 47.3 (46.9-47.7) | 46.4 (11.6) | 58.0 (15.5) | 46.4 (11.6) | 58.0 (15.5) |
| **Non-HDL-c, mg/dL** | 152.4 (151.3-153.5) | 152.4 (151.3-153.5) | 139.2 (30.9) | 131.5 (30.9) | 139.2 (30.9) | 135.3 (30.9) |
| **Statin Use, %** [c] | 8.5 (7.7-9.3) | 8.5 (7.7-9.3) | (17) | (14) | (17) | (14) |
| **Serum Glucose, mg/dL** [e] | 97.3 (96.7-98.0) | 97.3 (96.7-98.0) | --- | --- | --- | --- |
| **Anti-DM Medication, %** | 4.7 (4.3-5.1) | 4.7 (4.3-5.1) | --- | --- | --- | --- |
| **eGFR, mL/min per 1.73 m$^2$** | 99.3 (98.7-99.9) | 99.3 (98.7-99.9) | 91 (17) | 91 (19) | 91 (17) | 91 (18) |
| **Current/Former Smokers, %** [c, f] | 54.0 (52.3-55.6) | 54.0 (52.3-55.6) | (6.2) | (5.8) | (4.9) | (4.7) |
| **HTN, %** | 34.6 (33.3-36.0) | 35.2 (33.9-36.5) | --- | --- | --- | --- |
| **DM, %**[d] | 9.1 (8.4-9.8) | 8.8 (8.3-9.5) | (12) | (10) | (13) | (11) |
| **CKD, %** | 2.6 (2.3-2.9) | 4.9 (4.4-5.4) | --- | --- | --- | --- |

© 2024 Scheuermann B et al. *JAMA Network Open*.

[a] Data from the present study is presented as mean and 95% confidence interval. P.D. indicates PREVENT development cohorts, while P.V. indicates PREVENT validation cohorts. BMI: body-mass index; SBP: systolic blood pressure; DBP: diastolic blood pressure; HTN: hypertension; HDL: high-density lipoprotein; DM: diabetes mellitus; eGFR: estimated glomerular filtration rate; CKD: Chronic Kidney Disease.

[b] n=312 original values missing in the Present Study cohort.

[c] Only percentages (no 95% confidence intervals) were reported in the PREVENT publication.

[d] Total cholesterol, high-density lipoprotein, and non-HDL-c were converted from mmol/L in the PREVENT publication to mg/dL by multiplying by 38.67.

[e] n=1 original value missing.

[f] Percentages for the present cohort are combined current and former smokers; the PREVENT cohorts only reported current smokers.

© 2024 Scheuermann B et al. *JAMA Network Open*.

**eTable 4.** Reclassification table for only males from the overall cohort, created alongside net reclassification index analyses[a].

| 10-year Risk Estimated by PCE | 10-year Risk Estimated by PREVENT | | | | Total Counts[b] |
|---|---|---|---|---|---|
| | Low Risk (<5%) | Low-Moderate Risk (5-7.5%) | Moderate-High Risk (7.5-10%) | High Risk (≥10%) | |
| ***Participants who experienced a CVD Mortality Event*** | | | | | |
| **Low Risk (<5%)** | 7 (87.5) | 1 (12.5) | 0 (0) | 0 (0) | 8 |
| **Low-Moderate Risk (5-7.5%)** | 4 (50.0) | 2 (25.0) | 1 (12.5) | 1 (12.5) | 8 |
| **Moderate-High Risk (7.55-10%)** | 7 (77.8) | 2 (22.2) | 0 (0) | 0 (0) | 9 |
| **High Risk (≥10%)** | 5 (1.4) | 11 (3.1) | 9 (2.5) | 328 (92.9) | 353 |
| **Total Counts** | 23 | 16 | 10 | 329 | 378 |
| ***Participants who did not experience a CVD Mortality Event*** | | | | | |
| **Low Risk (<5%)** | 3921 (99.4) | 14 (0.4) | 4 (0.1) | 4 (0.1) | 3943 |
| **Low-Moderate Risk (5-7.5%)** | 692 (95.3) | 28 (3.9) | 5 (0.7) | 1 (0.1) | 726 |
| **Moderate-High Risk (7.55-10%)** | 517 (87.6) | 63 (10.7) | 7 (1.2) | 3 (0.5) | 590 |
| **High Risk (≥10%)** | 515 (13.4) | 646 (16.8) | 487 (12.7) | 2200 (57.2) | 3848 |
| **Total Counts** | 5645 | 751 | 503 | 2208 | 9107 |

[a] Entries in each cell are given as count (percentage, %) where the percentages are calculated as the cell count over the row sum.

[b] Note that the total counts do not sum to the total number of male participants in the cohort; this is because any subjects who did not experience an event but were censored before the timepoint of interest (10 years) are not included in analysis. Additionally, any subjects who experienced an event, but after the 10-year mark, are included in the counts for those who did not experience an event before 10 years.

© 2024 Scheuermann B et al. *JAMA Network Open*.

**eTable 5.** Reclassification table for only females from the overall cohort, created alongside net reclassification index analyses[a].

| 10-year Risk Estimated by PCE | 10-year Risk Estimated by PREVENT | | | | Total Counts[b] |
|---|---|---|---|---|---|
| | Low Risk (<5%) | Low-Moderate Risk (5-7.5%) | Moderate-High Risk (7.5-10%) | High Risk (≥10%) | |
| ***Participants who experienced a CVD Mortality Event*** | | | | | |
| **Low Risk (<5%)** | 19 (79.2) | 3 (12.5) | 2 (8.4) | 0 (0) | 24 |
| **Low-Moderate Risk (5-7.5%)** | 1 (16.7) | 4 (66.7) | 1 (16.7) | 0 (0) | 6 |
| **Moderate-High Risk (7.55-10%)** | 0 (0) | 3 (27.3) | 3 (27.3) | 5 (45.5) | 11 |
| **High Risk (≥10%)** | 0 (0) | 2 (0.6) | 4 (1.3) | 304 (98.1) | 310 |
| **Total Counts** | 20 | 12 | 10 | 309 | 351 |
| ***Participants who did not experience a CVD Mortality Event*** | | | | | |
| **Low Risk (<5%)** | 6755 (94.9) | 302 (4.2) | 41 (0.6) | 19 (0.3) | 7117 |
| **Low-Moderate Risk (5-7.5%)** | 232 (30.2) | 295 (38.4) | 163 (21.2) | 78 (10.2) | 768 |
| **Moderate-High Risk (7.55-10%)** | 87 (16.3) | 93 (17.4) | 192 (36.0) | 161 (30.2) | 533 |
| **High Risk (≥10%)** | 171 (8.0) | 42 (2.0) | 135 (6.3) | 1794 (83.8) | 2142 |
| **Total Counts** | 7245 | 732 | 531 | 2052 | 10560 |

[a] Entries in each cell are given as count (percentage, %) where the percentages are calculated as the cell count over the row sum.

[b] Note that the total counts do not sum to the total number of female participants in the cohort; this is because any subjects who did not experience an event but were censored before the timepoint of interest (10 years) are not included in analysis. Additionally, any subjects who experienced an event, but after the 10-year mark, are included in the counts for those who did not experience an event before 10 years.

© 2024 Scheuermann B et al. *JAMA Network Open*.

**eTable 6.** Demographic characteristics of the sub-cohort consisting of participants who did not have 'extreme' clinical variable values as described in the Methods[a].

| | Overall Sub-Cohort | Sub-Cohort, Males | Sub-Cohort, Females |
|---|---|---|---|
| **Sample Size, Unweighted n** | 21, 676 | 10,548 | 11,128 |
| **Sample Size, Weighted n (in millions)** | 153.2 | 75.7 | 77.5 |
| **Age, years** | 45.2 (44.8-45.6) | 44.2 (43.7-44.6) | 46.2 (45.7-46.7) |
| **Race/Ethnicity, %** [b] | | | |
| **Mexican-American** | 8.4 (7.1-9.8) | 9.1 (7.8-10.7) | 7.7 (6.4-9.1) |
| **Non-Hispanic Black** | 9.6 (8.4-10.9) | 9.2 (8.2-10.4) | 9.9 (8.6-11.4) |
| **Non-Hispanic White** | 71.4 (68.8-73.9) | 71.3 (68.7-73.7) | 71.5 (68.7-74.1) |
| **Other Hispanic** | 5.4 (4.2-7.0) | 5.3 (4.1-6.8) | 5.6 (4.3-7.2) |
| **Other/Multi-Racial** | 5.2 (4.5-6.0) | 5.1 (4.3-5.9) | 5.4 (4.6-6.2) |
| **BMI, kg/m$^2$** [c] | 27.5 (27.4-27.7) | 27.7 (27.6-27.9) | 27.4 (27.2-27.5) |
| **SBP, mmHg** | 121.7 (121.2-122.1) | 123.1 (122.7-123.6) | 120.2 (119.7-120.7) |
| **DBP, mmHg** | 71.2 (70.8-71.5) | 72.9 (72.6-73.3) | 69.4 (69.0-69.9) |
| **Anti-HTN Medication, %** | 17.7 (16.9-18.6) | 15.9 (14.9-17.0) | 19.5 (18.4-20.6) |
| **Total Cholesterol, mg/dL** | 201.9 (201.0-202.7) | 201.0 (200.0-202.0) | 202.7 (201.7-203.8) |
| **HDL, mg/dL** | 52.7 (52.4-53.1) | 47.4 (47.0-47.8) | 57.9 (57.4-58.4) |
| **Non-HDL-c, mg/dL** | 149.1 (148.3-150.0) | 153.5 (152.6-154.5) | 144.8 (143.7-146.0) |
| **Statin Use, %** | 7.9 (7.4-8.5) | 8.4 (7.7-9.2) | 7.5 (6.8-8.2) |
| **Serum Glucose, mg/dL**[d] | 94.8 (94.3-95.3) | 96.8 (96.1-97.5) | 93.0 (92.3-93.6) |
| **Anti-DM Medication, %** | 4.1 (3.8-4.4) | 4.2 (3.7-4.7) | 4.0 (3.6-4.5) |
| **eGFR, mL/min per 1.73 m$^2$** | 98.4 (97.8-99.0) | 98.9 (98.4-99.5) | 97.8 (97.1-98.5) |
| **Current/Former Smokers, %** | 47.7 (46.4-49.0) | 54.1 (52.4-55.7) | 41.5 (40.0-43.0) |
| **Hypertension, %** | 33.8 (32.7-34.9) | 33.5 (32.2-34.8) | 34.1 (32.8-35.4) |
| **Diabetes Mellitus, %** | 8.0 (7.6-8.5) | 8.3 (7.6-9.0) | 7.8 (7.3-8.4) |
| **CKD, %** | 3.7 (3.4-4.0) | 2.6 (2.3-2.9) | 4.8 (4.3-5.3) |

© 2024 Scheuermann B et al. *JAMA Network Open*.

[a] All values are presented as mean and 95% confidence interval. BMI: body-mass index; SBP: systolic blood pressure; DBP: diastolic blood pressure; HTN: hypertension; HDL: high-density lipoprotein; DM: diabetes mellitus; eGFR: estimated glomerular filtration rate; CKD: Chronic Kidney Disease.

[b] Other Hispanic included any self-reported Hispanic individuals who were not Mexican-American. The Other/Multi-racial category is not detailed further in NHANES methods.

[c] n=312 original values missing.

[d] n=1 original value missing.

© 2024 Scheuermann B et al. *JAMA Network Open*.

**eFigure 1.** Competing-risks receiver-operator characteristic curves demonstrating PREVENT sensitivity and specificity for cardiovascular and non-cardiovascular mortality specifically in male participants[a].

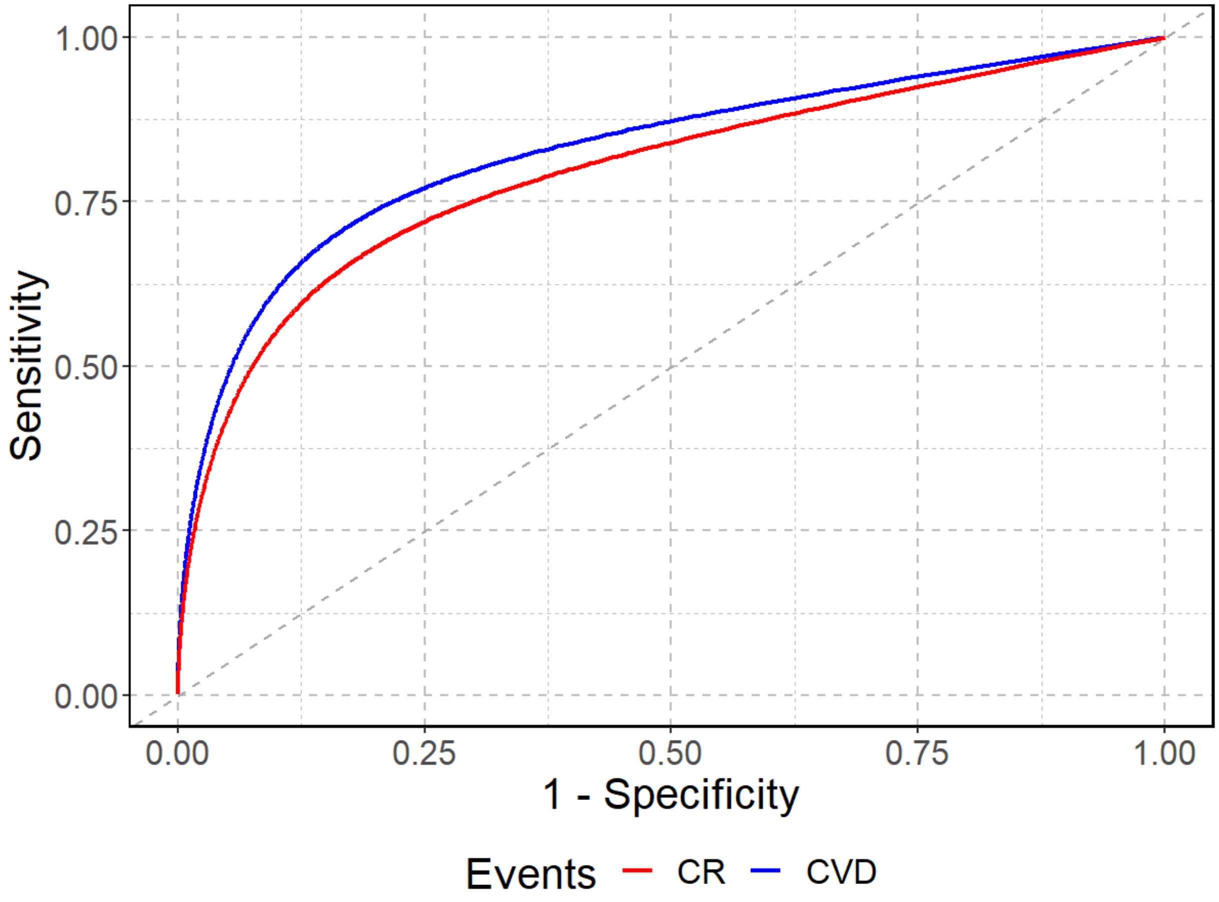


[a] CR indicates competing risk events (non-cardiovascular mortality events); CVD indicates cardiovascular mortality.

© 2024 Scheuermann B et al. *JAMA Network Open*.

**eFigure 2.** Competing-risks receiver-operator characteristic curves demonstrating PREVENT sensitivity and specificity for cardiovascular and non-cardiovascular mortality specifically in female participants[a].

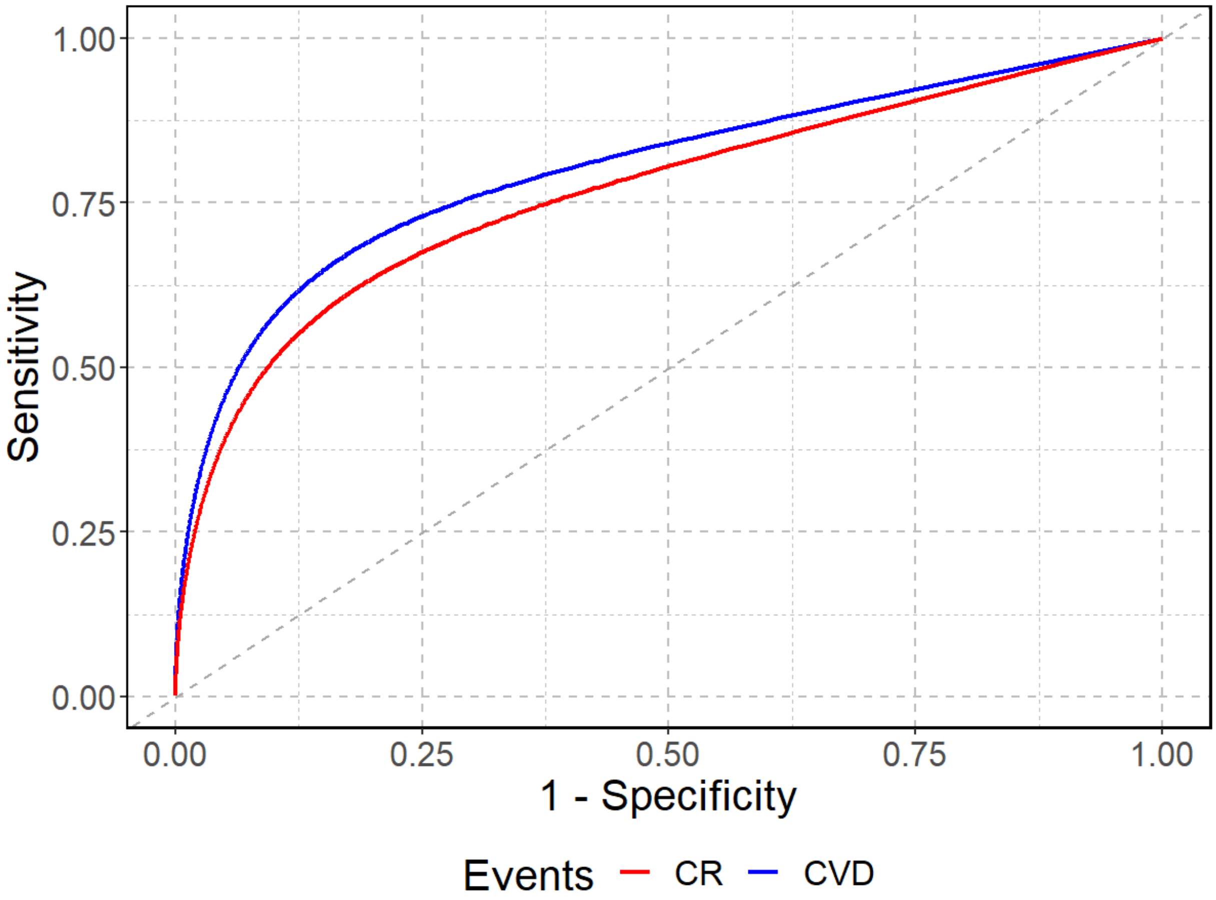


[a] CR indicates competing risk events (non-cardiovascular mortality events); CVD indicates cardiovascular mortality.

© 2024 Scheuermann B et al. *JAMA Network Open*.

**eFigure 3.** Competing-risks receiver-operator characteristic curves demonstrating PREVENT sensitivity and specificity for cardiovascular and non-cardiovascular mortality specifically after excluding participants who had 'extreme' clinical variable values[a].

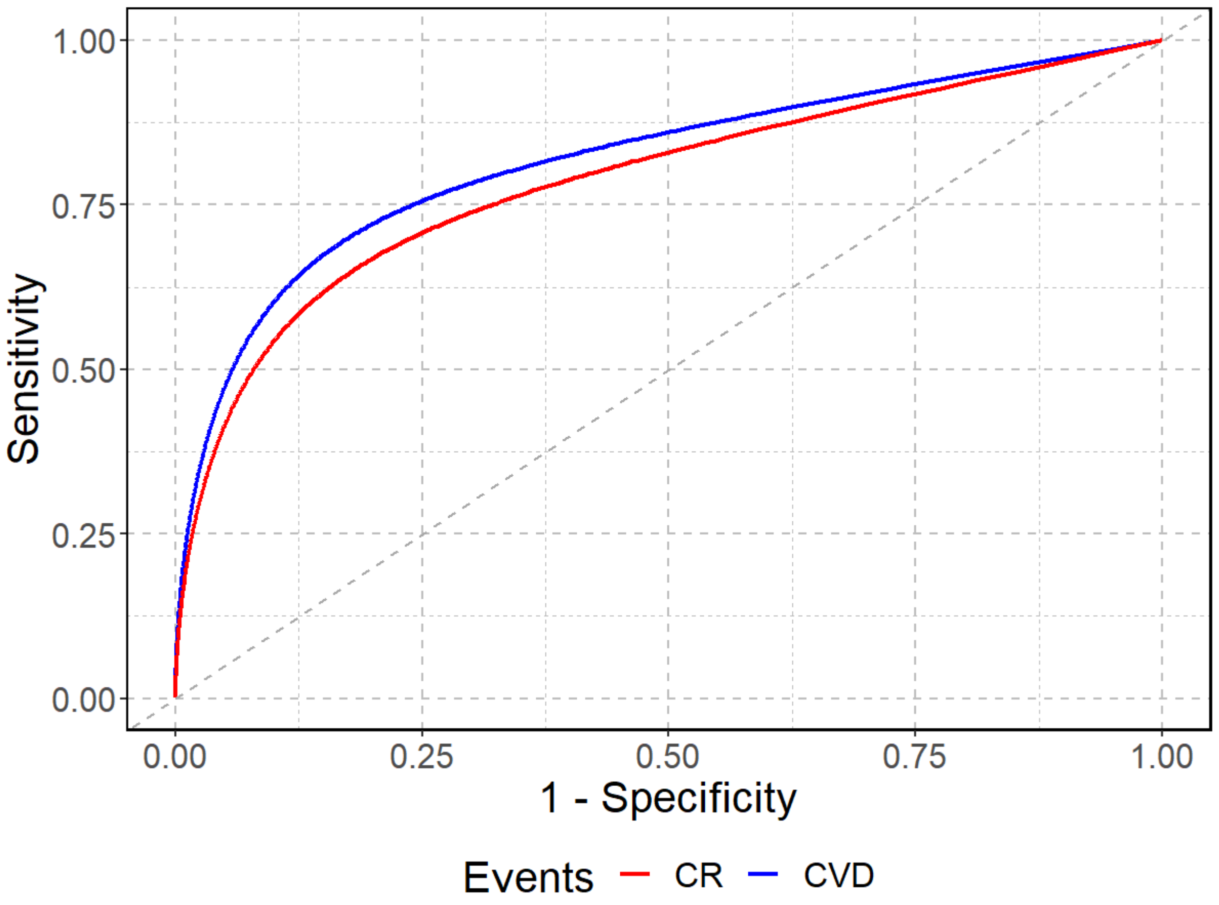


[a] CR indicates competing risk events (non-cardiovascular mortality events); CVD indicates cardiovascular mortality.

© 2024 Scheuermann B et al. *JAMA Network Open*.
