# Supplementary material for: External Validation of the American Heart Association PREVENT Cardiovascular Disease Risk Equations
Source: JAMA Netw Open. 2024 Oct 11;7(10):e2438311. doi: 10.1001/jamanetworkopen.2024.38311 (PMC11470385; doi:10.1001/jamanetworkopen.2024.38311)
Supplement: Supplement 2. — Data Sharing Statement [file jamanetwopen-e2438311-s002.pdf]

Data Sharing Statement

Scheuermann. External Validation of the American Heart Association PREVENT Cardiovascular Disease Risk Equations. *JAMA Netw Open*. Published October 11, 2024. doi:10.1001/jamanetworkopen.2024.38311

Data
Data available: Yes
Data types: Deidentified participant data
How to access data: http://wwwn.cdc.gov/nchs/nhanes/continuousnhanes/default.aspx
When available: With publication

Supporting Documents
Document types: None

Additional Information
Who can access the data: Anyone Requesting the Data
Types of analyses: For Any Purpose
Mechanisms of data availability: Without need for Investigator Support
